# Retrospective analysis of hospital electronic health records reveals unseen cases of acute hepatitis with unknown aetiology in adults in Oxfordshire

**DOI:** 10.1186/s12889-024-19292-1

**Published:** 2024-07-15

**Authors:** Cedric C. S. Tan, Gavin Kelly, Jack Cregan, Joseph D. Wilson, Tim James, Meera Chand, Susan Hopkins, Maaike Swets, J. Kenneth Baillie, Katie Jeffery, Ann Sarah Walker, David W. Eyre, Nicole Stoesser, Philippa C. Matthews

**Affiliations:** 1 The Francis Crick Institute, London, UK; 2 UCL Genetics Institute, University College London, London, UK; 3 Bioinformatics and Biostatistics, The Francis Crick Institute, London, UK; 4 Nuffield Department of Medicine, University of Oxford, Oxford, UK; 5 King’s College Hospital NHS Foundation Trust, London, UK; 6 Department of Biochemistry, Oxford University Hospitals NHS Foundation Trust, Oxford, UK; 7 United Kingdom Health Security Agency, Colindale, UK; 8 NIHR Health Protection Research Unit, Imperial College London, London, UK; 9 Leiden University Medical Center, Leiden, Netherlands; 10 Roslin Institute, University of Edinburgh, Edinburgh, UK; 11 Department of Infectious Diseases and Microbiology, Oxford University Hospitals, Oxford, UK; 12 Radcliffe Department of Medicine, University of Oxford, Oxford, UK; 13 Oxford University Hospitals NHS Foundation Trust, Oxford, UK; 14 Big Data Institute, Nuffield Department of Population Health, University of Oxford, Oxford, UK; 15 University College London Hospitals NHS Foundation Trust, London, UK; 16 University College London, London, UK

**Keywords:** Acute hepatitis, Adenovirus, Outbreak, AAV, Epidemiology, Electronic health records, Surveillance

## Abstract

**Background:**

An outbreak of acute severe hepatitis of unknown aetiology (AS-Hep-UA) in children during 2022 was subsequently linked to infections with adenovirus-associated virus 2 and other ‘helper viruses’, including human adenovirus. It is possible that evidence of such an outbreak could be identified at a population level based on routine data captured by electronic health records (EHR).

**Methods:**

We used anonymised EHR to collate retrospective data for all emergency presentations to Oxford University Hospitals NHS Foundation Trust in the UK, between 2016–2022, for all ages from 18 months and older. We investigated clinical characteristics and temporal distribution of presentations of acute hepatitis and of adenovirus infections based on laboratory data and clinical coding. We relaxed the stringent case definition adopted during the AS-Hep-UA to identify all cases of acute hepatitis with unknown aetiology (termed AHUA). We compared events within the outbreak period (defined as 1st Oct 2021—31 Aug 2022) to the rest of our study period.

**Results:**

Over the study period, there were 903,433 acute presentations overall, of which 391 (0.04%) were classified as AHUA. AHUA episodes had significantly higher critical care admission rates (*p* < 0.0001, OR = 41.7, 95% CI:26.3–65.0) and longer inpatient admissions (*p* < 0.0001) compared with the rest of the patient population. During the outbreak period, significantly more adults (≥ 16 years) were diagnosed with AHUA (*p* < 0.0001, OR = 3.01, 95% CI: 2.20–4.12), and there were significantly more human adenovirus (HadV) infections in children (*p* < 0.001, OR = 1.78, 95% CI:1.27–2.47). There were also more HAdV tests performed during the outbreak (*p* < 0.0001, OR = 1.27, 95% CI:1.17–1.37). Among 3,707 individuals who were tested for HAdV, 179 (4.8%) were positive. However, there was no evidence of more acute hepatitis or increased severity of illness in HadV-positive compared to negative cases.

**Conclusions:**

Our results highlight an increase in AHUA in adults coinciding with the period of the outbreak in children, but not linked to documented HAdV infection. Tracking changes in routinely collected clinical data through EHR could be used to support outbreak surveillance.

**Supplementary Information:**

The online version contains supplementary material available at 10.1186/s12889-024-19292-1.

## Introduction

In April 2022, the United Kingdom Health Security Agency (UKHSA) alerted the World Health Organization to a significant increase in acute severe hepatitis in children aged less than 10 years, who were otherwise clinically fit and well [1]. Concerningly, a proportion of these children had sufficiently severe disease to warrant liver transplantation [2]. Initial investigations and evaluation demonstrated no link to Hepatitis viruses A-E, other known causes of acute hepatitis, toxins, common exposures, or foreign travel; these cases were therefore designated ‘acute severe hepatitis of unknown aetiology’ (AS-Hep-UA).

Subsequent detailed investigation of samples from affected children suggested a likely infectious aetiology, with metagenomic sequencing identifying adeno-associated virus 2 (AAV2) in 81–96% AS-Hep-UA patients (versus 4–7% in controls), alongside a higher than expected prevalence of human adenovirus (HAdV) [3–5]. In addition to HAdV, a likely contribution was made by AAV coinfection with other ‘helper’ viruses including acute infections or reactivation of latent infections, particularly with Epstein-Barr Virus (EBV), human herpes-virus 6 (HHV6) and enteroviruses [3–5], and/or a contribution from superantigen-mediated immune activation [6]. A significant enrichment of the Human Leucocyte Antigen (HLA) class II allele DRB1*04:01 has been identified among AS-Hep-UA cases compared to the background population, suggesting a specific immune susceptibility [3].

Following the initial reporting of AS-Hep-UA in Scotland, several cases were retrospectively identified in the United States dating back to October 2021 [6]. By the start of July 2022, > 1000 probable cases had been identified worldwide [7]. The outbreak in Europe peaked between the end of March and early May 2022 (week 12 to 18), and subsequently declined between May and August [6]. The case definition of AS-Hep-UA was refined to include age < 16 presenting no earlier than October 1st 2021 with an acute hepatitis and deranged serum liver enzymes (alanine transaminase (ALT) or aspartate transaminase (AST) > 500 IU/L) which could not be accounted for by other causes [7].

Despite AS-Hep-UA being identified worldwide, there were geographical disparities in the incidence of cases and liver transplantation; rates in the UK and across parts of Europe clearly exceeded expected averages, in contrast to no significant deviation from baseline across the US, Brazil, India, and Japan [8, 9]. More than a quarter of global cases were identified in the UK, which had a 100-fold relative incidence rate compared to France, despite the countries being geographical neighbours of almost identical population sizes [10]. However, the relative contribution of enhanced surveillance, population susceptibility, and circulation of any causative agent to these differing rates has remained unclear. Furthermore, patients with acute hepatitis with unknown aetiology, but not meeting the stringent case definitions would not have been reported as AS-Hep-UA cases (i.e. those with ALT and/or AST elevated but both < 500 IU/L; age ≥ 16 years). Therefore, it is not known whether the AS-Hep-UA outbreak was the ‘tip of an iceberg’ of milder cases of disease in the population, and/or cases among older adolescents and adults.

Routinely collected clinical data (e.g. patient diagnoses, liver enzyme and microbiology test results) in the form of electronic health records (EHR) present an opportunity to investigate population trends that could be associated with this outbreak. There is potential to use routine clinical laboratory parameters as a surveillance tool at a population level, for example as a sentinel marker for circulation of an infectious trigger. In this study, we used hospital EHR data from Oxfordshire, UK, to explore trends before, during and after the period of the AS-Hep-UA outbreak. We addressed the following specific aims: (i) to explore any changes in liver enzyme levels in adults and children/adolescents presenting to hospital, and (ii) to determine any changes in incidence and severity of acute hepatitis and HAdV infection.

## Methods

### Data source

We analysed EHR representing children, adolescents and adults presenting as an emergency to Oxford University Hospitals (OUH) NHS Foundation Trust, a large tertiary referral hospital in the South East of England, serving a population of ~ 725,000. Data were accessed through the Infections in Oxfordshire Research Database (IORD) [11], and were held, accessed and analysed in accordance with NHS standards for data management and protection (more details in Supplementary methods).

In this retrospective cohort study, we reviewed data from 1st March 2016 to 31st December 2022 for all individuals aged 18 months and older presenting to the Emergency Department or acute medical/surgical assessment units at OUH. We recorded subsequent admission to hospital, admission to the Intensive Care Unit (ICU), duration of hospital admission, and mortality during the admission. Data from all individuals meeting these criteria were included in the analysis (we did not apply any exclusion criteria).

### Laboratory data

Laboratory data were generated by externally ISO accredited clinical biochemistry and microbiology laboratories at OUH. A full list of laboratory assays and platforms is provided in Suppl methods, and reference intervals for liver enzymes and inflammatory markers are provided in Table 1. Laboratory data were based on those routinely collected, where a request for ‘liver function tests’ (LFTs) prompts a clinical biochemistry profile comprising alanine transferase (ALT), alkaline phosphatase (ALP), bilirubin and albumin. Additional laboratory investigations were requested at the discretion of the clinical team. Abnormalities in these biomarkers were classified based on the upper limit of normal (ULN) for all ages and both sexes – mild, moderate and severe derangement was defined as up to 2x, 2–5 × and > 5 × ULN, respectively, with the exception of albumin, which was classified as deranged if levels were less than the lower limit of normal (LLN) of 32 g/L.

**Table 1:** Characteristics of population of adults and children aged ≥ 18 months presenting as an emergency to Oxford University Hospitals NHS Foundation Trust (UK) between 2016 and 2022

Characteristic			Younger children (18 months-6 years) *n* = 70,962	Older children/adolescents (7–15 years) *n* = 86,217	Adults (≥ 16 years) *n* = 746,254
Age in years at presentation (median, IQR)			3 (2–5)	11 (9–13)	53 (32–74)
Male sex (%)			57.1	54.2	47.5
Proportion admitted to hospital following emergency presentation (%)			17.0	13.2	34.3
Proportion admitted to ICU (%)			0.4	0.3	1.5
Mortality during admission episode (%)			0.01	0.01	0.41
Admission duration in hours if admitted, median (IQR)			20 (12–42)	24 (14–49)	56 (22–160)
	**Biomarker**	**Ref. Range**			
**Liver biomarkers** **(median, IQR)**	ALT	10–45 IU/L	15 (12–21)	14 (11–20)	19 (13–30)
	AST	15–42 IU/L	41 (31–144)	33 (21–119)	41 (22–110)
	Bilirubin	0–21 µmol/L	5 (3–7)	7 (5–11)	9 (6–14)
	Albumin	32–50 g/L	38 (35–40)	40 (38–43)	37 (33–40)
	ALP	30–130 IU/L	195 (160–237)	181 (115–247)	79 (64–101)
	GGT	15–40 IU/L	17 (10–104)	20 (13–62)	85 (31–280)
**Infection biomarkers** **(median, IQR)**	CRP	0–5 mg/L	10 (1.3–42.3)	2.5 (0.4–20.5)	8.6 (2.1–43.4)
	WBC count	4–11 × 10^9/L	10 (7.51–13.8)	8.53 (6.58–11.5)	8.87 (6.9–11.6)

*ALT* alanine transferase, *AST* aspartate transaminase, *ALP* alkaline phosphatase, *GGT* gamma glutaryl transferase, *CRP* C-reactive protein, *WBC* white blood cells

HAdV testing was undertaken using a PCR-based multiplex test on respiratory samples or using an HadV-specific PCR on whole blood based on specific clinician request, which usually focuses on patients requiring critical care or in immunocompromised patients under the care of haematology/oncology teams.

### Classification and definitions

Patients were stratified into three categories based on their ages at presentation: younger children (< 7 years), older children (7–15 years) and adults (≥ 16 years). Epochs were considered as pre-COVID-19 (1st March 2016—10 March 2020), COVID-19 pandemic period (11th March 2020—31st December 2022), and nested within the COVID-19 pandemic period, the AS-Hep-UA outbreak (1st Oct 2021—31 Aug 2022).

We applied the established strict case definition for AS-Hep-UA, as someone < 16 years of age presenting no earlier than 1st October 2021 with an acute hepatitis (ALT and/or AST > 500 IU/L), which cannot be accounted for by other causes [7]. We additionally applied a more relaxed definition of acute hepatitis of unknown aetiology (AHUA), to identify cases in adults, and also milder cases that would not meet criteria for AS-Hep-UA. We defined AHUA as patients assigned either a primary or secondary diagnostic code from the International Classification of Diseases 10th Revision consistent with hepatitis of an uncertain cause (Table 2; Supplementary Methods) or patients with ALT > 2 × ULN. Diagnostic codes were assigned by hospital admission coders following patient discharge, based on national clinical coding standards. We also considered presentations of diagnosed acute or chronic viral hepatitis A-E virus infection as a baseline control, and to ensure these cases were excluded from the AHUA category. We could not apply specific WHO criteria for acute liver failure, as this would require data regarding the duration of the liver injury (requiring liver function tests prior to the admission, and/or follow-up over time which is outside the scope of this analysis).

**Table 2:** Diagnostic codes representing Acute Hepatitis of Unknown Aetiology (AHUA) and confirmed viral hepatitis A-E infection assigned to patients presenting as an emergency to Oxford University Hospitals NHS Foundation Trust (UK) between 2016 and 2022. In order to protect against the risk of identification of individual cases, any subgroups numbering fewer than 5 individuals are not numerated

ICD10 code	Condition(s) coded	Younger children (18 months-6 years) *n* = 15	Older children (7–15 years) *n* = 12	Adults (≥ 16 years) *n* = 3702
**Codes representing acute hepatitis of unknown aetiology (AHUA; *n* = 1005)**				
K716	Toxic liver disease with hepatitis, not elsewhere classified	< 5	< 5	32
K720	Acute and subacute hepatic failure	< 5	< 5	534
K752	Nonspecific reactive hepatitis	< 5	< 5	< 5
K759	Inflammatory liver disease, unspecified	< 5	< 5	170
B178	Other specified acute viral hepatitis	< 5	< 5	13
B179	Acute viral hepatitis, unspecified	9	< 5	186
B199	Unspecified viral hepatitis without hepatic coma	< 5	< 5	46
**Codes representing viral hepatitis A-E (*n* = 2724)**				
B159	Hepatitis A	< 5	< 5	67
B162, B169, B180, B181	Acute or chronic viral hepatitis B ± delta virus	< 5	< 5	491
B171, B182	Acute or chronic viral hepatitis C	< 5	< 5	2057
B172	Acute viral hepatitis E	< 5	< 5	54

### Controls

We recorded cases of viral Hepatitis A-E as controls, as these infections are likely to be screened, diagnosed and recorded in clinical coding in individuals presenting with a clinical/laboratory picture of hepatitis, allowing us to assimilate baseline comparator data for infectious causes of elevated liver enzymes.

### Data sharing

Anonymised LFT data were shared with the Summary Analysis of Laboratory Tests (‘SALT’) project [12], co-ordinated by the UKHSA as part of the UK-wide public health response, to contribute to a national picture of changes in the incidence of deranged liver function (for epidemiology and ongoing surveillance). The complete datasets analysed during the current study are not publicly available as they contain personal data but are available from IORD, subject to the ethical and governance requirements of the database (details in Suppl methods). To protect anonymity, we avoided disaggregation into any category containing < 5 individuals.

### Data analysis and statistical testing

Each presentation episode was considered independently; thus individuals may have featured more than once across the study duration. We used the first set of blood tests taken on presentation for analysis. An infecting pathogen was reported if at least one microbiology test was positive. Data were analysed using *R* v4.1.2 and visualised using *ggplot* v3.4.0. The code used for all analyses is hosted on GitHub (https://github.com/cednotsed/iORD_hepatitis.git). We tested for the presence of a non-monotonic trend using the non-parametric WAVK test [13], using its implementation in the *R* package *funtimes* [14]. Fisher’s exact tests and Mann–Whitney U tests were performed using the *fisher.test* and *wilcox.test* functions in *R*. Odds ratios (OR) were calculated using conditional maximum likelihood estimation as part of the *fisher.test* function. An interupted time series analysis was performed to assess changes in the incidence of AHUA or viral hepatitis A-E during the study duration, using a segmented regression framework [15], as follows:

$${y}_{t}= {\beta }_{0}+ {\beta }_{1}t+{\beta }_{2}\left({\alpha }_{outbreak}\right)+ {\beta }_{3}(t-{t}_{start})+ {\varepsilon }_{t}$$

Where *y*_*t*_, *β*_*i*_, α_outbreak_, *t*_*start*_ and *ε*_*t*_ represent the incidence at time t, the parameter estimates, a binary variable encoding the AS-Hep-UA epoch, the start of the AS-Hep-UA epoch, and model residuals respectively. Autocorrelation and normality of *ε*_*t*_* was* assessed using the Durbin Watson test in the *lmtest R* package [16], and Shapiro–Wilk test, respectively. The statistical significance of parameter estimates was assessed using a Student’s t-test.

Associations between HAdV infection and routinely collected blood biomarker data were assessed using Fisher’s exact test. In particular, for each we tested if the proportion of patients falling into each derangement category (described above) differed significantly between patients with HAdV infections or otherwise. Benjamini–Hochberg procedure was used to correct for multiple testing and adjusted p-values, where available, were annotated.

## Results

### No evidence for an increase in hospital presentations or elevated liver enzymes during AS-Hep-UA outbreak

We analysed data for 903,433 acute hospital presentations, from 441,780 males and 461,632 females (and 21 individuals for whom sex was not recorded). The median age was 44 years (IQR 22–69 years), with 7.9%, 9.5%, and 82.6% classified as younger children, older children/adolescents, and adults, respectively (Table 1). A median of 11,023 patients presented to the hospital per month, with a marked decline in the number of presentations in April 2020 coinciding with the implementation of SARS-CoV-2 (COVID-19) pandemic lockdown measures in the UK introduced on 26th March 2020 (Supplementary Fig. 1a).

During the AS-Hep-UA outbreak, minimal changes in the number of acute presentations per month was observed across any of the three age groups (all WAVK tests *p* > 0.05; Supplementary Fig. 1a). There was an overall increasing trend in the number of ALT tests requested for acutely presenting patients over time since March 2016 regardless of sex (*WAVK statistic* = 13.436, *p* < 0.0001; Supplementary Fig. 1b). However, there was also an increasing trend in the number of ALT observations compared to WBC observations, which indicates increased ‘liver function scrutiny’ over time (Supplementary Fig. 1c; *WAVK statistic* = 91.127, *p* < 0.0001).

**Fig. 1:**
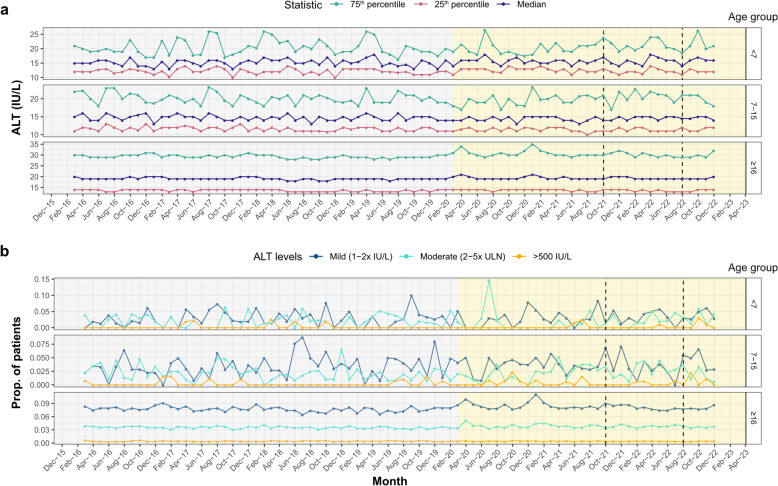
Temporal trends in ALT levels in patients presenting acutely to OUH (**a**) median, 25th and 75.^th^ percentiles of ALT levels in patients presenting to OUH and (**b**) proportion of patients with mild, moderate and severe derangement in ALT levels aggregated at 2 month intervals. Relevant epochs are highlighted in grey (pre-COVID-19-pandemic), yellow (COVID-19 pandemic), and with dashed lines (start of AS-Hep-UA outbreak to end of first quarter of 2022)

Across the study duration, 59% of patient episodes had recorded blood tests. Among these, 90% had an ALT test and 1.2% an AST test. There was no evidence of change in the median or IQR of ALT levels over time for any age group (Fig. 1a; WAVK tests *p* > 0.05), with no observable peak during the AS-Hep-UA outbreak. Similarly, the proportion of individuals with mild, moderate or severe derangement of ALT levels remained relatively stable over time (Fig. 1b; WAVK tests *p* > 0.05). Therefore, there was no temporal association between the period of the AS-Hep-UA outbreak and elevated liver enzymes in patients presenting acutely to hospital.

### Increased incidence of AHUA in adults coinciding with AS-Hep-UA outbreak

We further investigated the use of primary or secondary diagnostic codes for identifying increased incidence of AHUA in the patient population. We compared the temporal trend for AHUA diagnoses (Table 2; Supplementary Methods) against those for viral hepatitis A-E, the latter which are likely to be relatively stable and therefore serve as an appropriate control.

**Fig. 2:**
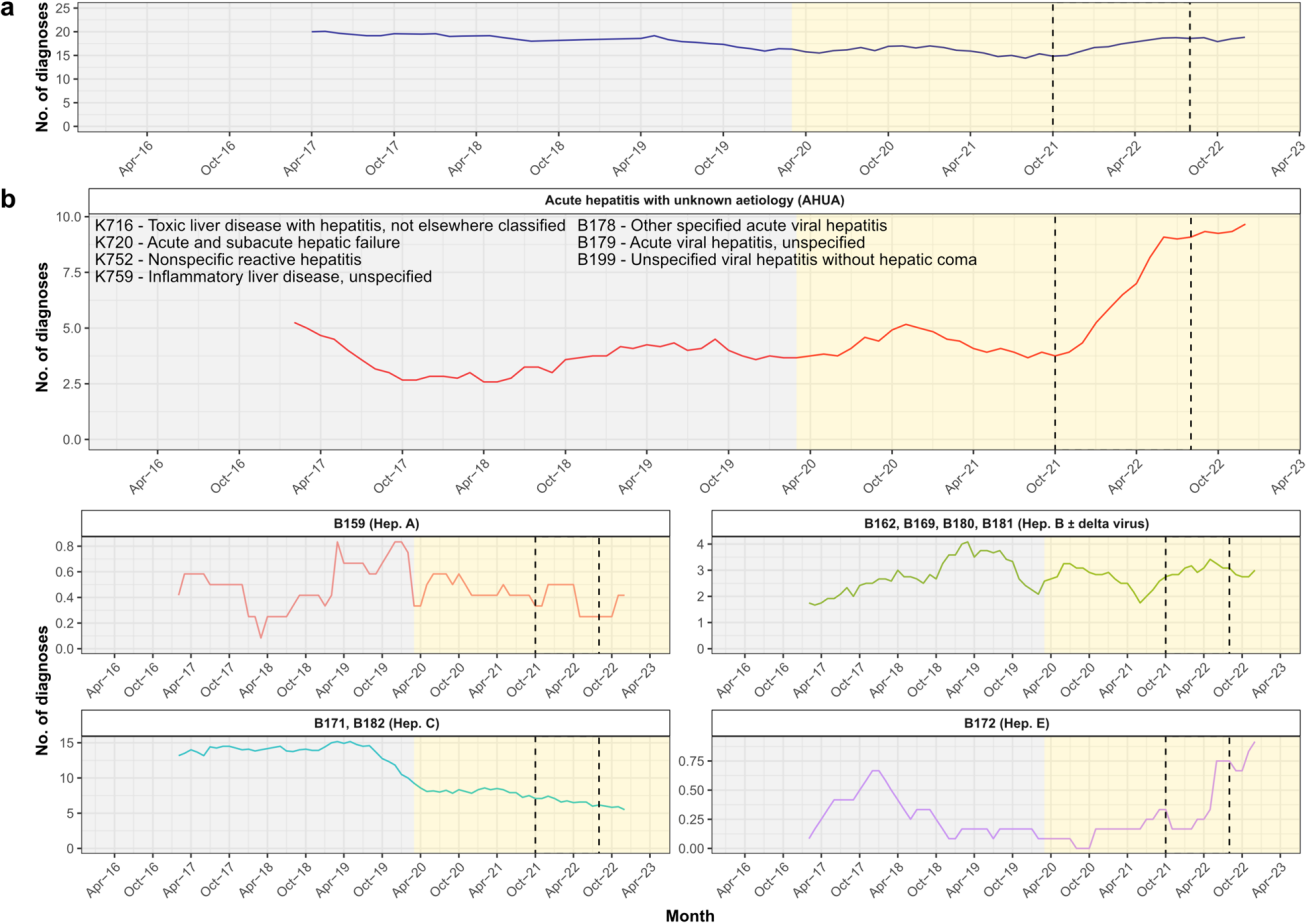
Temporal trends of acute hepatitis with unknown aetiology (AHUA) based on clinical coding at Oxford University Hospitals from 2016 to 2022. Twelve-month moving averages (means) of (**a**) overall number of hepatitis-related diagnoses (viral hepatitis A-E or AHUA) per month regardless of age group or sex, and (**b**) liver-related diagnoses with or without a specified causal agent (AHUA). ICD10 codes (primary or secondary) and their described causal agents are annotated. Codes are also expanded in Table 2. Relevant epochs are highlighted in grey (pre-COVID-19-pandemic), yellow (COVID-19 pandemic), and with dashed lines (start of AS-Hep-UA outbreak to end of first quarter of 2022)

Across the study duration, 3729 diagnostic codes representing AHUA (total 1005) or viral hepatitis A-E (total 2724) were assigned to 1531 distinct patient episodes (Table 2), of which 98% were adults (eight younger children, 11 older children/adolescents, 1512 adults). Overall, there were 391 patient episodes where only diagnostic codes classified as AHUA were assigned, representing 0.04% of all 903,433 patient episodes. The number of acute hepatitis diagnoses classified as AHUA or viral hepatitis A-E per month remained relatively constant over time (Fig. 2a). However, an increase in the number of AHUA cases coinciding with the AS-Hep-UA outbreak period was observed (Fig. 2b). The proportion of patients diagnosed with AHUA was higher during the AS-Hep-UA outbreak than outside this period (Fisher’s exact test *p* < 0.0001; OR 3.01, 95% CI:2.20–4.12).

Interrupted time series analysis indicated a significantly increased incidence of AHUA (estimate = 2.92, 95% CI: 1.57–5.69; t = 3.50, d.f. = 78, *p* < 0.001), but not viral hepatitis A-E (estimate = -0.785, 95% CI: -4.34–2.77; t = -0.440, d.f. = 78, *p* > 0.05), during the AS-Hep-UA outbreak period. Overall, these observations suggest an increased incidence of AHUA amongst adults during the AS-Hep-UA outbreak. We could not determine if this was the case for children, since only 2% of relevant diagnostic codes were assigned to children.

### AHUA associated with critical care admission, duration of hospitalisation and mortality

Compared to patients without AHUA, patients with AHUA had significantly higher ICU admission rates (Table 3; Fisher’s exact test *p* < 0.0001; OR 41.7 within and 23.7 outside the AS-Hep-UA epoch) and mortality rates (Fisher’s exact tests *p* = 0.035 within and *p* < 0.0001 outside; OR 6.99 within and 11.3 outside), and longer hospitalisations (both Mann–Whitney U tests *p* < 0.0001; Table 3). We also compared patients with AHUA to those diagnosed with viral hepatitis A-E; those with AHUA had significantly higher ICU admission rates (both Fisher’s exact test *p* < 0.0001; OR 5.01 within AS-Hep-UA outbreak and 3.90 outside outbreak) and longer hospitalisation periods (Mann–Whitney U tests *p* < 0.05), both within and outside the AS-Hep-UA epoch. Inpatient mortality was significantly higher for the AHUA group than those with viral hepatitis A-E outside the AS-Hep-UA epoch (Fisher’s exact test *p* < 0.0001; OR 19.8, 95% CI: 4.29–185), but not within the AS-Hep-UA epoch (*p* > 0.05; OR = 1.16, 95% CI: 0.0825–16.3).

**Table 3:** Outcomes of presentation to hospital among individuals presenting as an emergency to Oxford University Hospitals NHS Foundation Trust (UK) between 2016 and 2022

Population	Hospital admission rate (%)	ICU admission rate (%)	Duration of hospital admission in hours (median, IQR)	Mortality rate (%)
All patients (*n* = 903,433)	30.9	1.3	51 (21–146)	0.34
HAdV tested (*n* = 3707)	78.5	18.2	101 (45–221)	0.59
HAdV untested (*n* = 899,726)	30.7	1.2	50 (21–145)	0.34
HAdV tested and positive (*n* = 179)	60.9	11.7	65 (31–139)	0
HAdV tested and negative (*n* = 3528)	79.4	18.5	102 (46–224)	0.62
Patients with diagnostic codes relevant to the study (*n* = 1531)	100	12.3	93 (35–234)	1.1
Patients with only diagnostic codes indicative of AHUA (*n* = 391)	100	26.1	146 (57–333)	3.3
Patients with diagnostic codes indicating viral hepatitis A-E (*n* = 1140)	100	7.5	79 (29–199)	0.35
Patients without AHUA (903,042)	30.9	1.3	50 (21–145)	0.34

### Increased incidence of HAdV infections during AS-Hep-UA outbreak but not associated with deranged liver enzymes or poorer patient outcomes

Across the study duration, we retrieved 3707 distinct patient records that included microbiology tests for HAdV infection, of which 179 were positive (4.8%). The positivity rate was highest in younger children, among whom 124/781 (15.9%) of HAdV tests were positive, compared to older children/adolescents (9/440, 2.0% positive) and adults (46/2486, 1.9% positive), in keeping with the known epidemiology of HAdV infection [17–19]. None of the HAdV-infected patients were given ICD10 codes indicative of AHUA across the study duration. A minority (16/179; 9%) of HadV positive results were derived from eye swabs, which is unlikely to have influenced any overall trends.

**Fig. 3:**
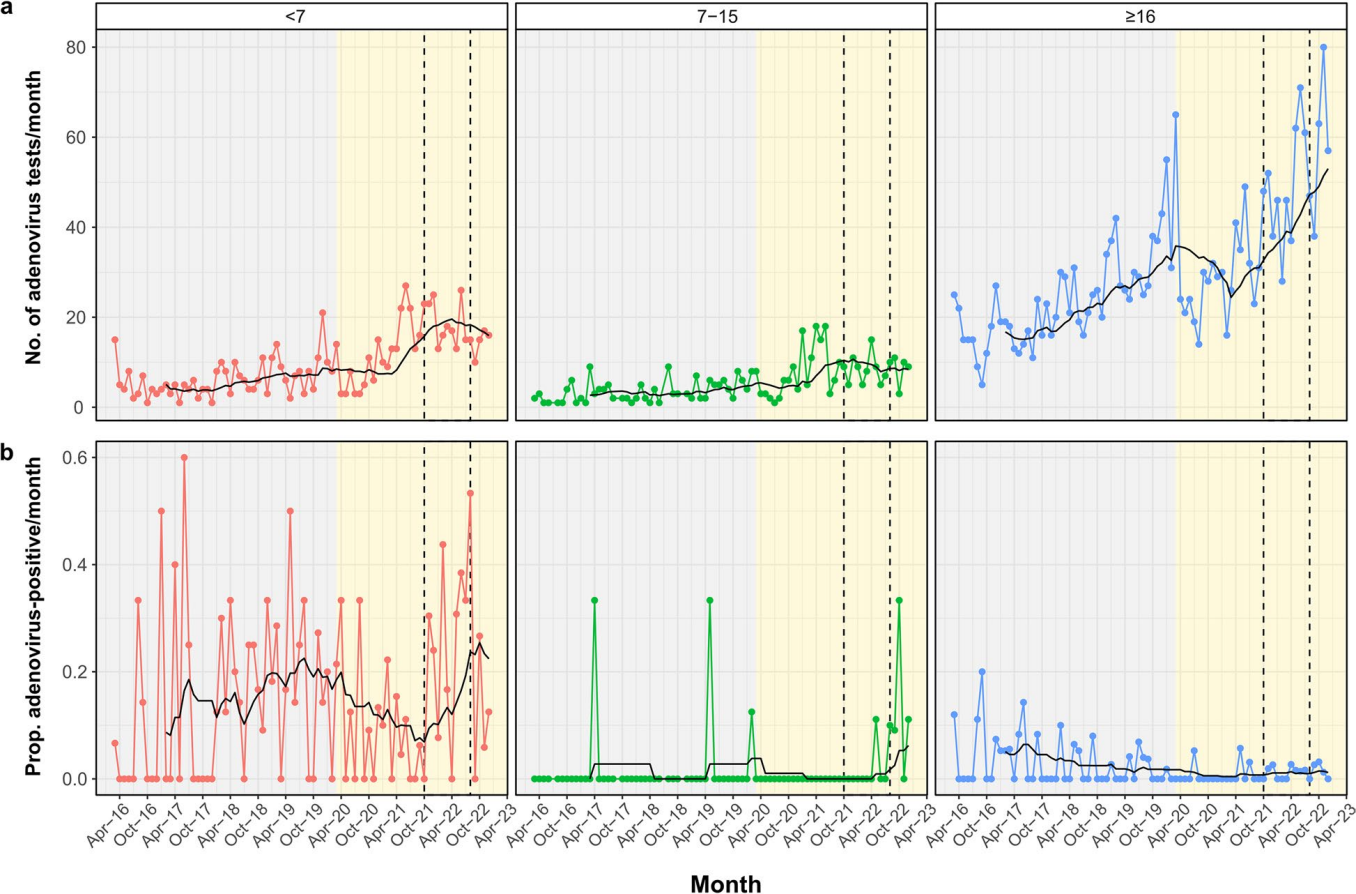
Temporal trends of HAdV-related microbiological tests requested at OUH from 2016 to 2022. **a** Number of HAdV tests requested and **b** the proportion of all HadV tests that were positive per month. Relevant epochs are highlighted in grey (pre-COVID-19-pandemic), yellow (COVID-19 pandemic), and with dashed lines (start of AS-Hep-UA outbreak to end of first quarter of 2022). Red, green, blue and black lines show data for for younger children (< 7 years), older children/adolescents (7–15 years), adults (≥ 16), and 12-month simple moving average, respectively

There was an increase in the number of HAdV-tests undertaken between April 2021 and April 2022 (Fig. 3a), and a significantly higher number of HAdV tests performed relative to all microbiology tests performed during the AS-Hep-UA epoch (Fisher’s exact test *p* < 0.0001; OR 1.27, 95% CI:1.17–1.37). These findings indicate increased clinician scrutiny for HAdV during the outbreak. The proportion of HAdV-positive tests during the AS-Hep-UA epoch was significantly higher than outside of the AS-Hep-UA epoch at 60/839 (7.2%) vs 119/2868 (4.1%) respectively (Fisher’s exact test *p* < 0.001; OR 1.78, 95% CI:1.27–2.47). Additionally, there was an increase in the incidence and proportion of HAdV-positive tests in younger children during the AS-Hep-UA outbreak relative to the period preceding the outbreak (Fig. 3b). However, there were also other peaks in the proportion of HAdV-positive tests across the entire study duration (Fig. 3b), indicating previous periods of high HAdV-positivity before the AS-Hep-UA epoch.

There was no evidence that the proportion of patients with mild, moderate or severe derangement of ALT, AST, bilirubin, GGT, CRP or WBC differed significantly between those testing positive vs. negative for HAdV (Fisher’s exact test *p* > 0.05; Supplementary Fig. 2). The proportion of patients with low albumin and mild derangement of ALP was significantly smaller for those testing HAdV-positive vs. negative (Fisher’s exact test *p* = 0.01 and *p* = 0.004 respectively; Supplementary Fig. 2). A similar association with raised ALP was also present in confirmed rhinovirus/enterovirus infections (Fisher’s exact test *p* = 0.004), indicating that mild derangement of ALP is not unique to HAdV infections (data not shown).

HAdV testing focuses primarily on a clinically vulnerable group, shown by higher rates of hospital admission, ICU admission and inpatient mortality among those receiving a HAdV test (irrespective of the test result) compared to the untested population (78.5% vs 30.7%, 18.2% vs 1.2%, and 0.59% vs 0.34%, respectively; Table 3). However, the HAdV-positive group fared somewhat better than those who tested negative, with lower hospital admission (both Fisher’s exact tests *p* < 0.001) and significantly shorter hospital stays (both Mann–Whitney U tests *p* < 0.05), whether within or outside the AS-Hep-UA epoch. Additionally, the HAdV-positive group had significantly lower ICU admission rates within the AS-Hep-UA epoch, but not outside the epoch (Fisher’s exact tests *p* = 0.015 and *p* = 0.35, respectively) (data not shown). No HAdV-positive patients died across the entire study duration. Characteristics of the population testing positive for HAdV are presented in Table 4.

**Table 4:** Characteristics of 179 patient episodes with HAdV-positive microbiology tests among children and adults presenting as an emergency to Oxford University Hospitals NHS Foundation Trust (UK) between 2016 and 2022

Characteristic		Younger children (18 months-6 years)	Older children (7–15 years)	Adults (≥ 16 years)
**Total number tested for HAdV infection**		781	440	2486
**Total number positive for HAdV infection (% of those tested)**		124/781 (15.9%)	9/440 (2.0%)	46/2486 (1.9%)
**Epoch of presentation**^**a**^	Pre-COVID-19 (*n* = 79 HAdV-positive)	45/79 (57.0%)	4/79 (5.1%)	30/79 (38.0%)
	COVID-19 pandemic (*n* = 100 HAdV-positive)	79/100 (79.0%)	5/100 (5.0%)	16/100 (16.0%)
	AS-Hep-UA (*n* = 60 HAdV-positive)	51/60 (85.0%)	2/60 (3.3%)	7/60 (11.7%)
**Levels of derangement for ALT**	Not tested	66	3	10
	Normal	53	5	25
	Mild (Up to 2 × ULN)	< 5	0	7
	Moderate (2–5 × ULN)	< 5	< 5	< 5
	Severe (> 5 × ULN)	0	0	< 5
**Inflammatory markers (median, IQR)**	CRP (mg/L)	47.2 (10.6–73.3)	24.0 (11.8–88.6)	81.9 (23.3–143.7)
	White cells (× 10^9/L)	11.6 (8.53–17.7)	9.1 (6.60–9.61)	10.4 (6.73–14.6)
**Admission rate**		74/124 (59.7%)	5/9 (55.6%)	30/46 (65.2%)
**ICU admission rate**		9/124 (7.3%)	0/9 (0%)	12/46 (26.1%)
**Duration of hospital admission in hours (median, IQR)**		56 (26–95)	118 (33–309)	125 (49–450)
**Death rate during admission**		0/124 (0%)	0/9 (0%)	0/46 (0%)
**Vital signs at presentation (median, IQR)**	Heart rate	134 (112–145)	111 (109–123)	92 (84–106)
	Diastolic blood pressure	61 (54.0–71.5)	81.5 (74.0, 86.3)	73.0 (61.3–82.0)
	Systolic blood pressure	104.0 (94.5–110.0)	109.0 (105.0–116.8)	123.5 (113.5–135.8)
	Tympanic temperature	37.5 (36.7–38.1)	37.3 (36.9–37.9)	36.9 (36.4–37.6)
	Oxygen saturation	98.0 (96.0–99.3)	98.0 (98.0–100.0)	96.0 (95.0–98.3)
	Respiratory rate	28.0 (24.0–35.0)	27.5 (22.5–30.5)	19.0 (17.0–21.0)

^a^AS-Hep-UA epoch is nested within the Covid-19 pandemic period. Epochs were considered as pre-COVID-19 (1st March 2016—10 March 2020), COVID-19 pandemic period (11th March 2020—31st December 2022), and the AS-Hep-UA outbreak (1st Oct 2021—31 Aug 2022)

## Discussion

We identified an increased incidence of episodes coded as AHUA in adults and an increased incidence of HAdV infections in younger children coinciding with the AS-Hep-UA outbreak in children during 2022. While the latter may be partially accounted for by increased clinician scrutiny during this period, the pattern was not observed to the same level in older children/adolescents, and not at all in adults despite similar increases in testing for all age groups. There was no evidence for increased incidence of abnormal liver enzymes in children or adults, nor associations between HAdV infections and elevated liver transaminases. Our findings suggest that the use of routinely collected liver enzyme EHR data lacks sensitivity for tracking this outbreak, which is likely due a large number of confounding aetiologies that may lead to elevated transaminases. However, the identification of increased incidence of AHUA in adults, which was largely ignored during the AS-Hep-UA outbreak in children, highlights the potential of using hospital diagnostic codes for cost-effective disease surveillance.

Among patients presenting acutely for hospital-based care, HAdV testing is largely reserved for vulnerable groups. Even in this high-risk population, those testing positive for HAdV had lower admission rates than those testing negative, reinforcing the view that this virus is generally benign and self-limiting, with a low risk of serious complications. The lack of associations between HAdV infections and deranged liver enzymes is concordant with the fact that HAdV infections typically lead to mild respiratory or gastrointestinal disease, and that hepatitis is an unusual complication [19]. Co-infections with HAdV and other viruses such as respiratory syncytial virus (RSV) have been linked to poorer outcomes [19], but the small number of HAdV infections identified in this study precluded robust analysis of mixed infections. Despite an increased incidence of HAdV infections during the AS-Hep-UA outbreak, this was not an unusual aspect of local HAdV epidemiology, and could have been partly accounted for by increased clinician scrutiny.

It has emerged that severe clinical outcomes during the AS-Hep-UA outbreak in children was likely driven by AAV2 as a leading aetiological agent. However, AAV2 requires co-infection with a ‘helper’ virus (including, but not limited to, HAdV) to replicate, and this appears to be a requirement for the development of liver pathology [3–5]. Retrospective analysis of EHRs cannot be used to investigate the epidemiology of AAV infection, as these viruses are not part of clinical diagnostic testing pathways and have only been identified as an agent of AS-Hep-UA through retrospective metagenomic sequencing [3–5]. The specific subtype of HAdV implicated in the outbreak, 41F, is not routinely discriminated from other types by clinical testing. Other possible ‘helper viruses’ include human herpesviruses [5], many of which are ubiquitous in the population and characterised by long-term carriage and latency, making it difficult to distinguish between clinically relevant episodes and subclinical reactivation. Thus routinely-collected clinical datasets do not include screening for all relevant pathogens, and detection of implicated pathogens can be difficult to interpret due to the detection of commensal or bystander organisms. Overall, wider adoption of metagenomics-based diagnostics has the potential to further enhance the utility of EHRs to investigate future outbreaks but interpretation is complex [4].

Although we have captured data for a large population over a period of almost seven years, this remains a small data set within which to identify rare events, and our region was not known to be directly affected by the AS-Hep-UA outbreak. Measurement of liver enzymes is a blunt approach to the identification of liver disease, and abnormalities reflect diverse pathology. In the intensive care population, some cases meeting our criteria for AHUA may be related to liver ischaemia or injury from diverse causes associated with critical illness, rather than liver-specific pathology. Hospital coding data varies in its accuracy, and the sensitivity and specificity of codes used to identify cases of hepatitis are not known, but may be low, particularly when the aetiology has not been confirmed.

There are various caveats to our approach for detection of AHUA. If there were milder cases of disease in the population during or preceding the documented AS-Hep-UA outbreak, these may not have presented to hospital at all. Even among those presenting to healthcare, many patients did not have liver enzymes measured. Thus, the IORD dataset provides only a limited view of the whole population, which is not true community surveillance. Hospital admission data are also biased by repeated representation of the same individuals, and over-represent populations who preferentially present to emergency care rather than accessing primary care, or those admitted through different routes. As we did not include children < age 18 months, we may have missed relevant signals in the younger population. Clinical datasets are always subject to missingness, which may not be random. Detection of relevant pathogens also depends on the sample type collected. Our analyses also relies on consistent clinical coding of patient episodes, which is potentially subject to some inaccuracy and/or variability.

Anonymised clinical data from EHRs offers access to large datasets, providing power in numbers to determine overall trends reflecting clinical epidemiology and its influence on morbidity, mortality and health service workload. Monitoring of EHRs may be an effective and low-cost surveillance tool that allows identification of trends that could be of concern—e.g. deranged laboratory parameters and/or changes in recorded diagnoses based on microbiology tests or coding. Such strategies could potentially be developed to provide an ‘early warning’ system to allow clinical and public health authorities to review data in real time, cross-compare between regions, identify possible outbreaks, and implement enhanced surveillance and public-health messaging if necessary. As we have demonstrated, analysis of EHRs allowed us to identify an increased incidence of AHUA amongst adults, which was not determined during the AS-Hep-UA outbreak and warrants further investigation. Longer-term collation of data from multiple regions would offer a more powerful approach that could be extended to other diseases. However, surveillance through EHRs requires the establishment of suitable and systematic data-processing infrastructure and governance frameworks, in addition to investment of personnel and resources, if it is to become a real-world surveillance tool.

### Supplementary Information

Supplementary Material 1.

## Data Availability

The data that support the findings of this study are available from the IORD database but restrictions apply to the availability of these data, which were used under license for the current study, and so are not publicly available. The IORD database can only be accessed by named investigators in accordance with NHS standards for data management and protection. For further details on how to apply for access to the data and for a research proposal template please email iord@ndm.ox.ac.uk. All custom scripts used for the analyses presented in this manuscript are hosted on GitHub (https://github.com/cednotsed/iORD_hepatitis.git).

## References

[1] World Health Organization, Disease Outbreak News: Acute hepatitis of unknown aetiology – the United Kingdom of Great Britain and Northern Ireland. 2022. https://www.who.int/emergencies/disease-outbreak-news/item/2022-DON368#.

[2] Morfopoulou S, Buddle S, Montaguth OET, Atkinson L, Guerra-Assunção JA, Marjaneh MM (2023). Genomic investigations of unexplained acute hepatitis in children. Nature.

[3] Ho A, Orton R, Tayler R, Asamaphan P, Herder V, Davis C (2023). Adeno-associated virus 2 infection in children with non-A-E hepatitis. Nature.

[4] Servellita V, Gonzalez AS, Lamson DM, Foresythe A, Huh HJ, Bazinet AL (2023). Adeno-associated virus type 2 in US children with acute severe hepatitis. Nature.

[5] Matthews PC, Campbell C, Săndulescu O, Matičič M, Ruta SM, Rivero-Juárez A, et al. Acute severe hepatitis outbreak in children: A perfect storm. What do we know, and what questions remain? 2022. 10.3389/fphar.2022.1062408 PMC9732095 36506522

[6] Kambhampati AK (2022). Trends in acute hepatitis of unspecified etiology and adenovirus stool testing results in children—United States, 2017–2022. MMWR Morb Mortal Wkly Rep.

[7] Zhang L-Y, Huang L-S, Yue Y-H, Fawaz R, Lim JK, Fan J-G (2022). Acute hepatitis of unknown origin in children: early observations from the 2022 outbreak. J Clin Transl Hepatol.

[8] van Beek J, Fraaij PL, Giaquinto C, Shingadia D, Horby P, Indolfi G (2022). Case numbers of acute hepatitis of unknown aetiology among children in 24 countries up to 18 April 2022 compared to the previous 5 years. Eurosurveillance.

[9] European Centre for Disease Prevention and Control report; Increase in severe acute hepatitis cases of unknown aetiology in children. https://www.ecdc.europa.eu/en/increase-severe-acute-hepatitis-cases-unknown-aetiology-children.

[10] Oxford Biomedical Research Centre Infections in Oxford Rsearch Database (IORD). https://oxfordbrc.nihr.ac.uk/research-themes/modernising-medical-microbiology-and-big-infection-diagnostics/iord-about/.

[11] International Severe Acute Respiratory and emerging Infection Consortium (ISARIC). Summary Analysis of Laboratory Tests (SALT). https://isaric4c.net/salt/.

[12] Lyubchich V, Gel YR, El-Shaarawi A (2013). On detecting non-monotonic trends in environmental time series: a fusion of local regression and bootstrap. Environmetrics.

[13] Lyubchich V, Gel YR, Brenning A, Chu C, Huang X, Islambekov U, Niamkova P, Ofori-Boateng D, Schaeffer ED, Vishwakarma S, Wang X. Package ‘funtimes’, Functions for Time Series Analysis. 2023. https://cran.r-project.org/web/packages/funtimes/funtimes.pdf.

[14] Lynch III JP, Kajon AE. Adenovirus: Epidemiology, global spread of novel types, and approach to treatment. vol. 42, Thieme Medical Publishers, Inc.; 2021, p. 800–21. 10.1055/s-0041-1733802 34918322

[15] Cooper R, Hallett R, Tullo A, Klapper P (2000). The epidemiology of adenovirus infections in greater Manchester, UK 1982–96. Epidemiol Infect.

[16] Sandkovsky U, Vargas L, Florescu DF (2014). Adenovirus: current epidemiology and emerging approaches to prevention and treatment. Curr Infect Dis Rep.

[17] Lenaerts L, De Clercq E, Naesens L (2008). Clinical features and treatment of adenovirus infections. Rev Med Virol.

[18] Probst V, Spieker AJ, Stopczynski T, Stewart LS, Haddadin Z, Selvarangan R (2022). Clinical presentation and severity of adenovirus detection alone vs adenovirus co-detection with other respiratory viruses in US children with acute respiratory illness from 2016 to 2018. J Pediatr Infect Dis Soc.

[19] Magiorkinis G, Matthews PC, Wallace SE, Jeffery K, Dunbar K, Tedder R (2019). Potential for diagnosis of infectious disease from the 100,000 genomes project metagenomic dataset: recommendations for reporting results. Wellcome Open Res.

