# Supplementary material for: Retrospective analysis of hospital electronic health records reveals unseen cases of acute hepatitis with unknown aetiology in adults in Oxfordshire
Source: BMC Public Health. 2024 Jul 15;24:1890. doi: 10.1186/s12889-024-19292-1 (PMC11251388; doi:10.1186/s12889-024-19292-1)
Supplement: Supplementary file 1 — Supplementary Material 1. [file 12889_2024_19292_MOESM1_ESM.docx]

**SUPPLEMENT**

**Retrospective analysis of hospital electronic health records**

**reveals unseen cases of acute hepatitis with**

**unknown aetiology in adults in Oxfordshire**

Cedric Tan et al.

**Supplementary methods**

1. **The Infections in Oxfordshire Research Database (IORD)**

The Infections in Oxfordshire Research Database (IORD) has ethical approvals from the National Research Ethics Service South Central – Oxford C Research Ethics Committee (19/SC/0403), the Health Research Authority and the national Confidentiality Advisory Group (19/CAG/0144), including provision for use of pseudonymised routinely collected data without individual patient consent. Individuals who choose to opt out of their data being used in research are not included in the study. The study was carried out in accordance with all relevant guidelines and regulations. The study sponsor was OUH. All patients were assigned an anonymised ‘cluster ID’, with no identifying details handled by the research team. Only month/year of birth was available rather than specific date of birth. Data were held within a password protected, encrypted database and accessed only by named investigators in accordance with NHS standards for data management and protection.

More details can be found at the following URL:

<https://oxfordbrc.nihr.ac.uk/research-themes-overview/antimicrobial-resistance-and-modernising-microbiology/infections-in-oxfordshire-research-database-iord/>),

For further details on how to apply for access to the data and for a research proposal template please email [iord@ndm.ox.ac.uk](mailto:iord@ndm.ox.ac.uk).

1. **ICD10 codes**

**Codes used for AHUA:**

K759, K752, K720, K716, B178, B179, and B199 (also see **Table 2** in the main text**).**

**Codes used to identify acute or chronic viral hepatitis (hepatitis A-E viruses)**

B159, B162, B169, B180, B182, B171, B182, B172 (listed as either primary or secondary diganostic codes).

**Laboratory tests and assay platforms**

Details of liver function testing (LFTs) are presented in the main text. Additional tests were performed at the discretion of the clinical team, as follows:

- Liver function tests, standard panel comprises alanine transferase (ALT), alkaline phosphatase (ALP), bilirubin and albumin; expanded panel performed on specific clinical request adds aspartate transaminase (AST) and gamma glutamyl transferase (GGT): standard methods on Abbott Architect c16000 analysers (Abbott Laboratories, Maidenhead, UK).
- C-reactive protein (CRP): standard methods on Abbott Architect c16000 analysers (Abbott Laboratories, Maidenhead, UK).
- Full blood count (FBC) including a white blood cell count (WBC): Sysmex XN automated analyser (Sysmex UK Ltd., Milton Keynes, UK).
- Human adenovirus (HadV) testing:
  - Respiratory panel: (FilmArray Respiratory 2.1 [RP2.1] Panel, BioFire Diagnostics)
  - Blood and eye samples: Adenovirus ELITe MGB® Kit, ELITech Group SpA (real-time PCR).

**Supplementary figures**

**Supplementary Figure 1. Temporal trends in presentations to hospital and laboratory requests per month over the time period of the study**. (a) Number of patients presenting to hospital, (b) number of ALT tests requested, and (c) ratio of ALT to WBC tests requested. Relevant epochs are highlighted in grey (pre-COVID-19-pandemic), yellow (COVID-19 pandemic), and with dashed lines (start and end of AS-Hep-UA outbreak to end of first quarter of 2022).

**
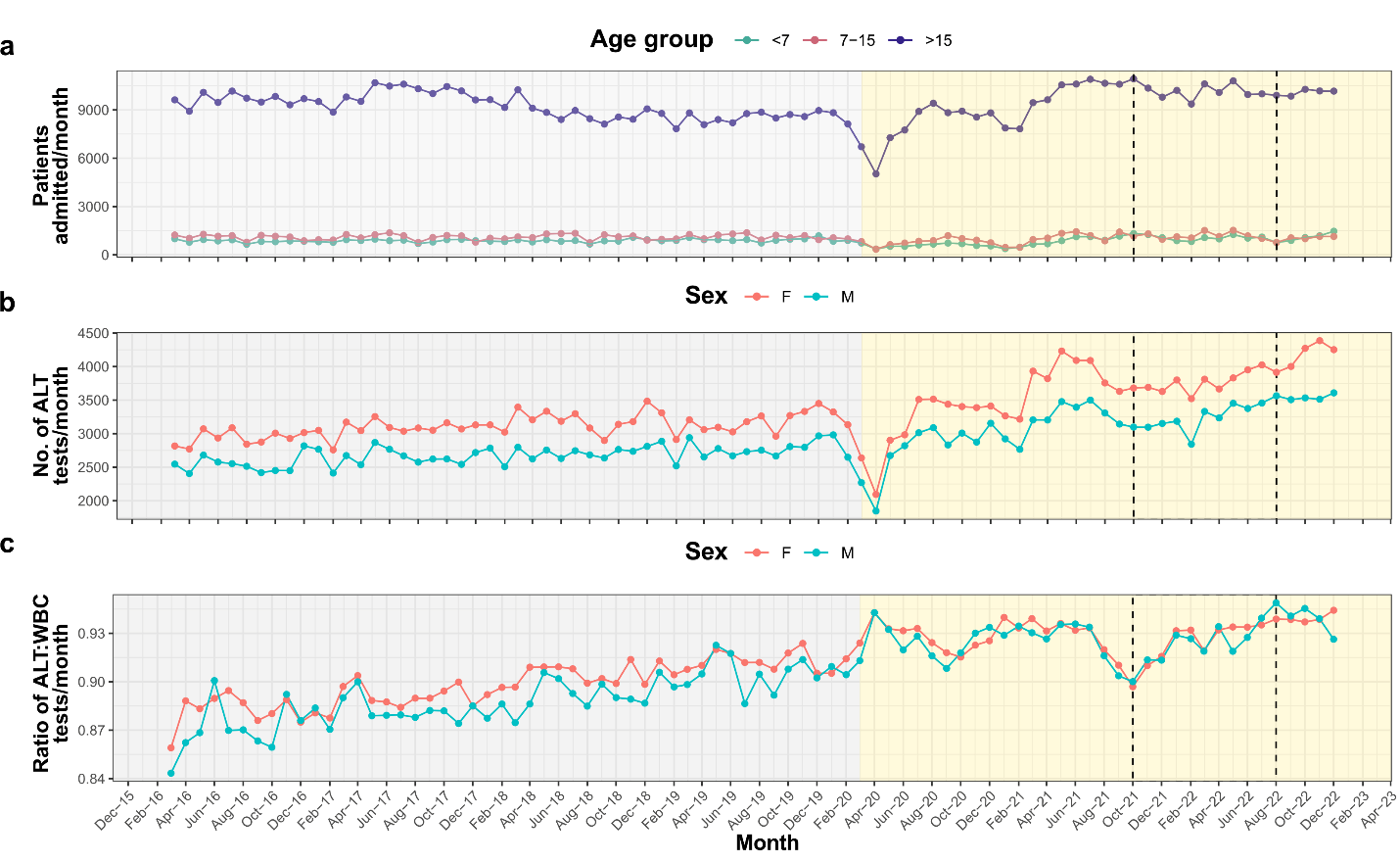
**

**Supplementary Figure 2. Associations between HAdV** **infection and blood biomarkers**. Barchart showing the proportion of patients, stratified by HAdV infection, with various levels of derangement of blood biomarkers. Albumin levels were considered deranged if they were less than the lower limit of normal (32g/L). For each blood biomarker, Fisher’s exact test was used to determine if the proportion of patients falling into each derangement category differed significantly between patients with HAdV infections or otherwise. Benjamini-Hochberg procedure was used to correct for multiple testing and adjusted p-values, where available, were annotated.

**
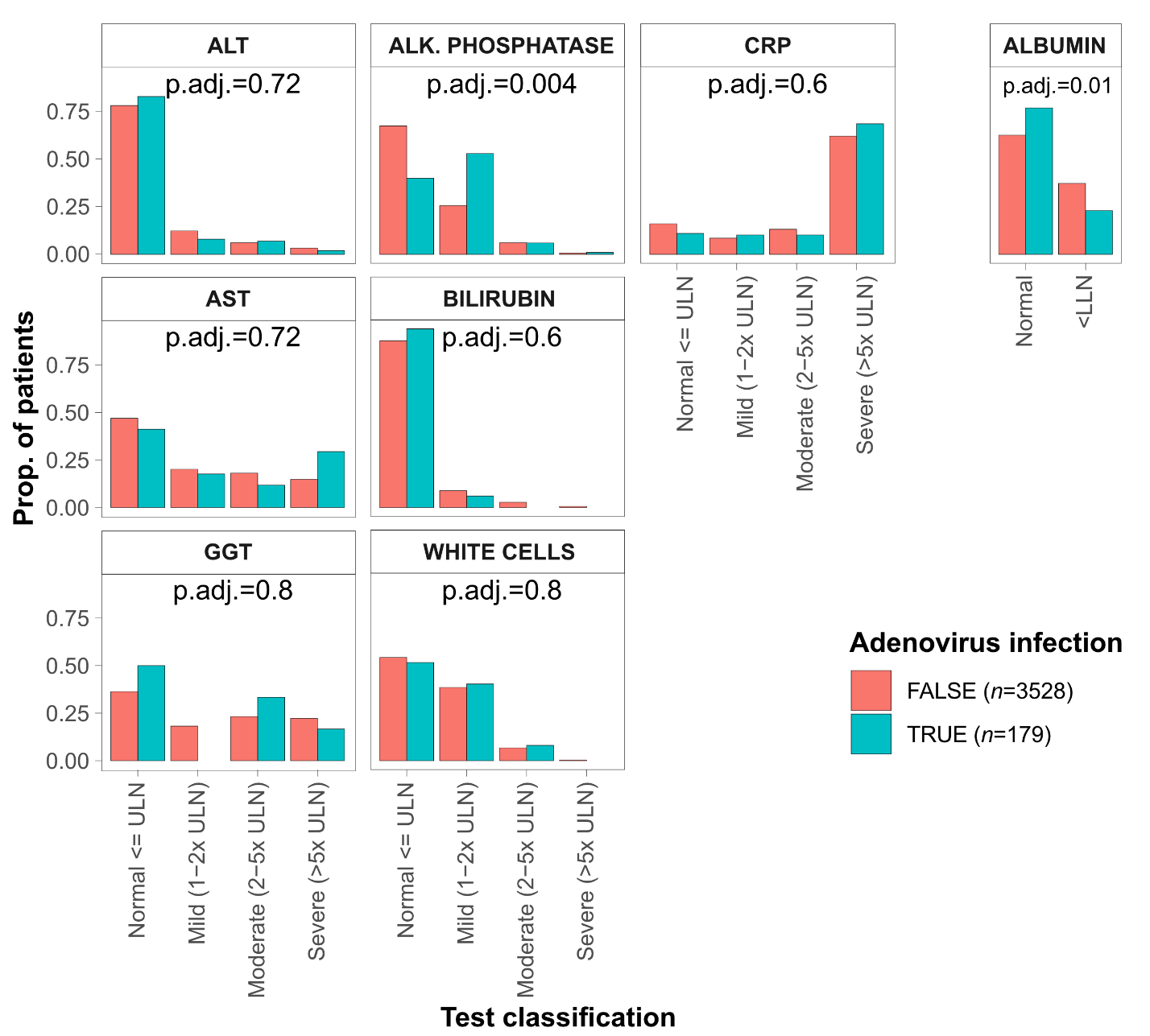
**
